# A Novel Square-Root Cubature Information Weighted Consensus Filter Algorithm for Multi-Target Tracking in Distributed Camera Networks

**DOI:** 10.3390/s150510526

**Published:** 2015-05-05

**Authors:** Yanming Chen, Qingjie Zhao

**Affiliations:** Beijing Key Laboratory of Intelligence Information Technology, School of Computer Science, Beijing Institute of Technology, Beijing 100081, China; E-Mail: zhaoqj@bit.edu.cn

**Keywords:** cubature Kalman filter, information filter, consensus algorithm, multi-target tracking, distributed camera networks

## Abstract

This paper deals with the problem of multi-target tracking in a distributed camera network using the square-root cubature information filter (SCIF). SCIF is an efficient and robust nonlinear filter for multi-sensor data fusion. In camera networks, multiple cameras are arranged in a dispersed manner to cover a large area, and the target may appear in the blind area due to the limited field of view (FOV). Besides, each camera might receive noisy measurements. To overcome these problems, this paper proposes a novel multi-target square-root cubature information weighted consensus filter (MTSCF), which reduces the effect of clutter or spurious measurements using joint probabilistic data association (JPDA) and proper weights on the information matrix and information vector. The simulation results show that the proposed algorithm can efficiently track multiple targets in camera networks and is obviously better in terms of accuracy and stability than conventional multi-target tracking algorithms.

## 1. Introduction

With the rapid development of image processing, sensor and semiconductor technology, the availability of inexpensive hardware, such as CMOS cameras, that are able to ubiquitously capture video content from the environment has fostered the development of camera networks [1]. Cameras have been widely used in smart homes, wide-area surveillance, intelligent transportation, medical care, industrial control, *etc*.

Multiple cameras can cover a large area, communicate with each other through the network and then fuse all of their measurements to achieve robust scene understanding. However, factors, such as weather, illumination and shadow, make the measurements suffer noise easily. At the same time, there are multiple targets in the scene, which increases the difficulty of targets tracking. In this paper, we focus on the problem of tracking multi-targets through a camera network. In many application scenarios of camera networks, the observation is a nonlinear function of the target state. Consequently, we propose a novel algorithm for these complicated application scenarios.

A camera network is a set of resource-constrained camera-equipped sensor nodes that are spread over a large area. The limit of the centralized architecture is obvious. When there are large volumes of data that need to be transmitted, processed and interpreted by resource-constrained nodes to deliver to the fusion center, the network may easily fail because of the energy consumption and communication burden.

One way of addressing this issue is through the novel paradigm of distributed algorithms. Recently, distributed algorithms have witnessed a surge in interest that has enabled a wide range of cooperation and information fusion in bandwidth-limited sensor networks. They are advantageous for target tracking in camera networks due to their scalability and high fault tolerance [2,3].

In a distributed estimation scheme, the system must adopt certain strategies to share information. In recent years, many researchers have proposed linear consensus protocols to deal with this problem through multiple iterations of communication between the local node and its neighboring nodes. For example, Olfati-Saber *et al.* [4] provided a theoretical framework for consensus and cooperation in multi-agent systems. In their paper, they made a detailed analysis of a consensus algorithm for multi-agent networked systems with an emphasis on the role of directed information flow, robustness to changes in the network topology due to link/node failures, time delays and performance guarantees. Ren *et al.* [5] considered the problem of information consensus among multiple agent exchange with dynamically changing interaction topologies and gave conditions for asymptotic consensus under dynamically changing interaction topologies and the weighting factors using update schemes.

Combining with the above-mentioned consensus algorithm and then using a filter algorithm, such as Kalman filter, one can achieve the goal of target tracking. Olfati-Saber introduced a novel distributed Kalman consensus filtering (KCF) algorithm for sensor networks [6]. The KCF algorithm works under the assumption that every sensor has the ability to sense all targets. However, in a realistic camera network, a target could usually be seen by none or only a few of the cameras. In [7], Olfati-Saber *et al.* considered the case mentioned above. However, the solution is a hybrid P2P/hierarchical architecture, not fully distributed and not suitable for large-scale networks. Kamal *et al.* [2] proposed an information weighted consensus filter (IWCF) to deal with this problem by proper weights on the prior state and the measurement information. In camera networks, the measurement model does not evolve linearly. Hence, tracking algorithms depending on linear filters, such as the traditional Kalman filter and the information filter, cannot be applied. Katragadda *et al.* [8] proposed two consensus-based distributed algorithms for nonlinear systems using the extended information filter (EIF). However, this filter adopts multivariate Taylor series expansions to linearize a model. The accuracy may not meet the requirements when they are used in the case of camera networks.

To solve these problems, this paper proposes a novel consensus filter based on the square-root cubature Kalman filter (SCKF) [9]. The SCKF adopts a third-degree spherical-radial cubature rule that provides a set of cubature points scaling linearly with the state-vector dimension. The SCKF can provide a robust and systematic solution for high-dimensional nonlinear filtering problems. Meanwhile, compared with the unscented Kalman filter (UKF) [10], the SCKF can preserve two properties of the error covariance matrix: symmetry and positive definiteness in each update cycle [9]. In the UKF, due to errors introduced by arithmetic operations performed on finite word-length digital computers, these two properties are often lost.

One advantage of the information filter over the Kalman filter arises from its natural fit for multi-agent problems. Multi-agent problems often involve the integration of sensor data collected decentrally. Such integration is commonly performed using Bayes' rule. When represented in logarithmic form, Bayes' rule becomes an addition. Information integration is achieved by summing up information from multiple sensors. Addition is commutative. Because of this, information filters often integrate the information in an arbitrary order, with arbitrary delays and in a completely decentralized manner [11]. In this paper, we use the information form of SCKF, which is called the square-root cubature information filter (SCIF) [12].

Multi-target tracking is the combination of data association and estimation. However, the above-mentioned methods do not consider the measurement-to-track association. Among many algorithms that are available for data association, the multiple hypothesis tracking (MHT) [13] and joint probabilistic data association (JPDA) [14] are two popular schemes. JPDA achieves reasonable results at a much lower computational cost than MHT and can be easily integrated into a distributed system.

The main contribution of this paper is proposing data association with a square-root cubature information filter, taking special care of the issues of nonlinearity and finite word-length digital computers and using the proposed algorithm to track multi-targets in a camera network. In Section 2 the state-of-the-art in distributed multi-target tracking in camera networks is described. Section 3 presents preliminaries for this paper, such as the model, average consensus and JPDA. In Section 4, the distributed square-root cubature information weighted consensus filter (DSCIWCF) is proposed. We describe the JPDA with DSCIWCF for the multi-target tracking algorithm, called the multi-target square-root cubature information weighted consensus filter (MTSCF), in Section 5. In Section 6, the proposed method is compared against others experimentally. The simulation results show that the proposed algorithm can efficiently track multiple targets in camera networks. Finally, we will give the conclusion of this paper in Section 7.

## 2. Related Work

This section discusses consensus-based distributed multi-target tracking in camera networks, focusing on the problems of nonlinearity, redundancy and robustness.

There are many research papers on multi-target tracking under sensor networks [15–20]. However, most of these methods do not consider the problem of naive nodes [2] and numerical difficulties resulting from the finite word-length of computers. Now that computers have become so much more capable, we do not have to worry about numerical problems as before. Nevertheless, numerical issues still arise in finite-word-length implementations of algorithms, especially in sensor networks.

In [15], a distributed data association for multi-target tracking in sensor networks was proposed by Sandell *et al.* In their paper, they considered that each sensor node can make noisy measurements of the target state. In this situation, data association techniques must be employed. Therefore, they used the JPDA algorithm to deal with the data association. Although their proposed method is distributed, their method is based on KCF, which is used in a linear system and does not consider naive nodes.

In [18], Roy-Chowdhury *et al.* extended the method proposed in [15] to deal with nonlinear problems. Although it can be used in nonlinear camera networks, their method is based on EKCF, and thus, naive nodes have not been considered.

Kamal proposed extended multi-target information consensus to deal with the problems of nonlinearity and naive nodes [20]. Their method is based on IWCF [2], which is more robust and accurate than the KCF algorithm. However, their method has the problem of numerical difficulties mentioned above in resource-constrained camera networks.

As described above, this paper uses JPDA with the SCIF-based tracking algorithm at each camera node to track multi-targets in a camera network. Our algorithm can not only overcome the numerical difficulties mentioned above, but also gets much more accurate results at the same time.

## 3. Basic Theories

### 3.1. System Model

The general nonlinear system model for camera networks is the form:

(1) $$x_{k,i}^{j}=f(x_{k-1,i}^{j})+v_{k-1,i}^{j}$$

(2) $$z_{k,i}^{j}=h(x_{k,i}^{j})+w_{k,i}^{j}$$

where the system equation *f*(·) and the measurement equation *h*(·) are time-varying nonlinear functions. At time *k*, $x_{k,i}^{j}\in {\text{R}}^{n_{x}}$ is the state vector of the *j*-th target. Each camera *C_i_* gets *m_i_*(*k*) measurements denoted as ${\left\{z_{k,i}^{j}\right\}}_{j=1}^{m_{i}(k)}$, and $z_{k,i}^{j}\in {\text{R}}^{n_{z}}$ is the nonlinear measurement from the *j*-th target measured by the node *C_i_* at time *k*. Cameras do not know the relationship between measurements and targets. That is to say, they do not know which measurement is generated from which target. $v_{k-1,i}^{j}\in {\text{R}}^{n_{x}}$ is the process noise of the node *C_i_* on time *k* − 1, $w_{k,i}^{j}\in {\text{R}}^{n_{z}}$ is the measurement noise of node *C_i_* at time *k*. The noise sequences $v_{k-1}^{i}$ and $w_{k}^{i}$ are assumed to be independent and white with $v_{k-1}^{i}\sim \text{N}(0,Q_{k-1}^{i})$ and $w_{k}^{i}\sim \text{N}(0,R_{k}^{i})$, respectively.

Given a camera network with *N_C_* cameras, there are no specific assumptions about the overlap among the FOVs of these cameras. In the FOV, there are *N_T_* moving targets. In this paper, we assume that all cameras have been calibrated, so we can get the target position corresponding to the same reference plane. The communication in the network can be represented using an undirected connected graph *G*(τ) = (*C*, *E*(τ), *A*(τ)) [21,22]. The set of vertices *C* = {*C*_1_,*C*_2_,…,*C_N_C__*} represents the cameras. The set *E* ⊆ *C* × *C* contains the edges of the graph, which represents the available communication channels between different cameras. *A*(τ) = [*a_ij_*]_*N*_*C*_×*N*_*C*__ is an adjacency matrix, which is a symmetric 01—matrix. Because the graph has no loops, the diagonal entries of *A*(τ) are zero (a*_ii_* = 0, *i* = 1,…, *N_C_*). Ω*_i_* = {*j* ∈ *C* | (*i*,*j*) ∈ *E*} is an adjacency set of node *C_i_*. (*i,j*) represents the direct communication channel between node *C* and node *C_j_*. The degree of node *C* is the number of its neighbors Δ*_i_* = Σ*_j_a_ij_*. The degree matrix is an *N_C_* × *N_C_* matrix defined as Δ(*t*) = *diag*{*A*(τ) • 1}. The Laplacian of graph *G*(τ) is defined by *l*(τ) = Δ(τ) − *A*(τ).

In this paper, we use the “+” superscript to denote the *a posteriori* estimate and the “–” superscript to denote the *a priori* estimate. For example, ${\hat{x}}_{k,i}^{j-}$ (and its covariance $P_{k,i}^{j-}$) represents the prior/predicted state estimate (and covariance) of $x_{k,i}^{j}$.

### 3.2. Average Consensus

To compute the average, average consensus [23,24] is a popular distributed algorithm. Suppose, each node *i* holds an initial scalar value *a_i_*(0) ∈ **R**, and $\text{a}(0)={\left\{a_{i}\right\}}_{i=1}^{N_{C}}$ denotes the vector of the initial node values on the network. We are interested in computing the average of the initial values, $\frac{1}{N_{C}}\sum_{i=1}^{N_{C}}a_{i}$ via a distributed algorithm, in which the nodes only communicate with their neighbors.

In the average consensus algorithm, at the beginning of iteration τ, a node *C_i_* sends its previous state *a_i_*(τ − 1) to its direct network neighbors *C_j_* ∈ Ω*_i_* and also receives the neighbors' previous states *a_j_* (τ − 1). Then, the iterative form of the average consensus algorithm can be stated as follows in discrete-time:

(3) $$a_{i}(\tau )=a_{i}(\tau -1)+є\sum_{j\in \Omega_{i}}\left(a_{j}(\tau -1)-a_{i}(\tau -1)\right)$$

By several iterations, a consensus is asymptotically reached for all initial states. The rate parameter *є* should be chosen in 0 ~ 1/Δ*_max_*, where Δ*_max_* is the maximum degree of the network graph *G*. Choosing a larger value of *є* will result in faster convergence, but choosing values equal or more than 1/Δ*_max_* will render the algorithm unstable. The paper [24] provided a good choice of є using Metropolis weights. The Metropolis weight matrix is defined as:

(4) $$W_{ij}=\{\begin{array}{ll} \frac{1}{1+\max\{\Delta_{i},\Delta_{j}\}}, & \text{if}\;\{i,j\}\in E\\ 1-\sum_{\{i,k\}\in E}W_{ik}, & \text{if}\;i=j\\ 0, & \text{otherwise} \end{array}$$

Arranging the local consensus states into the vector $\text{a}[\tau ]=[a_{1}^{T}[\tau ],\cdots ,a_{N_{C}}^{T}[\tau ]]$, the update Equation (3) can be written in the matrix form as:

(5) a[τ+1]=(Ψ[τ]⊗I)a[τ]

where Ψ[τau;] = **I** — є**L**(τ), **I** is the appropriate size identity matrix and ⊗ denotes the matrix Kronecker product; Ψ[τ] is a stochastic matrix.

### 3.3. Joint Probabilistic Data Association

In the real world, in addition to the data originating from the target, a set of measurements are clutter, which correspond to no targets. A direct measurement to target assignment may lead to poor performance. Thus, a data association algorithm is needed. In this paper, we use JPDA for data association [14,15]. Here, we briefly review this algorithm.

The idea of JPDA is to compute the smoothing property of expectations. In other words, the conditional mean of the state is obtained by averaging over all of the association events. Let $\beta_{ij}^{t}=P[\chi_{ij}^{t}|Z_{i}^{k}]$ and $\beta_{i0}^{t}=1-\sum_{j=1}^{m_{i}(k)}P[\chi_{ij}^{t}|Z_{i}^{k}]$. β*_i_*_0_ denotes the probability that no measurement is associated with target *t* for node *C_i_*, and $\chi_{ij}^{t}$ denotes the event that the measurement *j* on node *i* originated from target *t*. See [14] for details about computing β*_ij_*'s values. The JPDA filter (JPDAF) state estimate is:

(6) $$\begin{array}{ll} {\hat{x}}_{i}^{t+} & =E[x_{t}|Z_{i}^{k}]\\ & ={\hat{x}}_{i}^{t-}+K_{i}^{t}(z_{i}^{t}-(1-\beta_{i0}^{t})H_{i}^{t}{\hat{x}}_{i}^{t-}) \end{array}$$

where ${\hat{x}}_{i}^{t+}$ and ${\hat{x}}_{i}^{t-}$ denote the *a posteriori* estimate and prior estimate of the state of target *t* by node *i* at time *k*, respectively. $z_{i}^{t}$ and $K_{i}^{t}$ denote the mean measurement and the Kalman gain for target *t*, respectively. $H_{i}^{t}$ is the observation matrix for node *C_i_* for target *t*. *Z_i_*(*k*) = {*z_i_*_1_(*k*), *z_i_*_2_(*k*),⋯} denotes the set of *m_i_*(*k*) measurements obtained by node *i* at time *k*, and we define $Z_{i}^{k}=\{Z_{i}(1),Z_{i}(2),\ldots Z_{i}(k)\}$.

From Equation (6), the mean measurement innovation ${\tilde{z}}_{i}^{t}$ for target *t* is defined as:

(7) $${\tilde{z}}_{i}^{t}=z_{i}^{t}-(1-\beta_{i0}^{t})H_{i}^{t}{\hat{x}}_{i}^{t-}$$

where $z_{i}^{t}=\sum_{j=1}^{m_{i}(k)}\beta_{ij}^{t}z_{ij}$.

The covariance estimate for JPDAF is given by:

(8) $$P_{i}^{t+}=P_{i}^{t-}-(1-\beta_{i0}^{t})K_{i}^{t}W_{i}^{t}{\left(K_{i}^{t}\right)}^{T}+K_{i}^{t}{\tilde{P}}_{i}^{t}{\left(K_{i}^{t}\right)}^{T}$$

where:

(9) $${\tilde{P}}_{i}^{t}=\sum_{j=1}^{m_{i}(k)}\beta_{ij}^{t}(z_{ij}-H_{i}^{t}{\hat{x}}_{i}^{t-}){\left(z_{ij}-H_{i}^{t}{\hat{x}}_{i}^{t-}\right)}^{T}-({\tilde{z}}_{i}^{t}){\left({\tilde{z}}_{i}^{t}\right)}^{T}$$

The JPDAF is based on the Kalman filter, which is the best linear estimator. However, in this paper, the camera model is a nonlinear system. The JPDAF needs to be modified to fit the nonlinear system. Details will be discussed in Section 5.

## 4. Square-Root Cubature Information Weighted Consensus Filter

The square-root cubature Kalman filter (SCKF) algorithm [9] was proposed by Arasaratnam *et al.* It is a more accurate nonlinear filter that could be applied to solve high-dimensional nonlinear filtering problems with minimal computational effort. In multi-sensor data fusion applications, because of the advantages of the information filter mentioned above, this paper uses the information square-root cubature information filter (SCIF) [12]. Firstly, we will give a brief review of SCIF of node *i* as follows; thus, in order to facilitate the description, the sensor index *i* will be dropped in this review.

### 4.1. Square-Root Cubature Information Filter: A Brief Review

The information filter propagates the inverse of *P*, rather than propagating *P*. The state estimate and its corresponding covariance in the Kalman filter are replaced by the information vector and information matrix, respectively, in the information filter. The updated information vector and information matrix can be written as:

(10) $$Y_{k|k-1}=P_{k|k-1}^{-1}=S_{y,k|k-1}S_{y,k|k-1}^{T}$$

(11) $${\hat{y}}_{k|k-1}=P_{k|k-1}^{-1}{\hat{x}}_{k|k-1}=Y_{k|k-1}{\hat{x}}_{k|k-1}$$

where *S_y,k_*_|_*_k_*_−1_ is the square-root information matrix. The information update at time *k* is given by:

(12) $$Y_{k|k}=Y_{k|k-1}+I_{k}$$

(13) $${\hat{y}}_{k|k}={\hat{y}}_{k|k-1}+i_{k}$$

Here, *I_k_* and *i_k_* are defined as follows, respectively [12]:

(14) $$I_{k}=(Y_{k|k-1}P_{xz,k|k-1})R_{k}^{-1}{\left(Y_{k|k-1}P_{xz,k|k-1}\right)}^{T}$$

(15) $$i_{k}=(Y_{k|k-1}P_{xz,k|k-1})R_{k}^{-1}(z_{k}-{\hat{z}}_{k|k-1}+P_{xz,k|k-1}^{T}{\hat{y}}_{k|k-1})$$

In matrix theory, an covariance matrix *P* can be written as:

(16) $$P=AA^{T}$$

where *P* ∈ **R***^n^*^×^*^n^, A* ∈ **R**^*n* × *m*^, *m* ≥ *n*. Equation (16) can be considered as the square-root of *P*. For a simple calculation, in this paper, *A* is transformed into a *n* × *m* triangular matrix *S* using a triangularization decomposition algorithm, as follows:

(17) $$S=\text{Tria}(A),S\in {\text{R}}^{n\times m}$$

Tria denotes a triangularization decomposition algorithm. If we use QR decomposition, A*^T^* will be decomposed into an orthogonal matrix *Q* ∈ **R***^m^*^×^*^m^* and an upper triangular matrix *R* ∈ **R***^m^*^×^*^n^*, *A^T^* = *QR*; then, Equation (16) can be written as:

(18) $$P=AA^{T}=R^{T}Q^{T}QR=R^{T}R=SS^{T}$$

then *S* = *R^T^*. *S* is a lower triangular matrix, which is a sparse matrix. The sparsity of *S* will benefit calculations and reduce storage space. The steps involved in the square-root cubature information filter algorithm are summarized in the following:

In the time update, at time *k*, assume that (*ŷ_k_*_−1|_*_k_*_−1_, *S_y,k_*_−1|_*_k_*_−1_) is known; we compute the square-root of the predicted information matrix *S_y,k_*_|_*_k_*_−1_ and the predicted information vector *ŷ_k_*_|_*_k_*_−1_.
In the measurement update, we will compute the updated information vector *ŷ_k_*_|_*_k_* and the square-root of the updated information matrix *S_y,k_*_|_*_k_* according to the results of the time update step. See [15] for details about these two steps.

### 4.2. Centralized Square-Root Cubature Information Filter

Multi-sensor fusion is the process by which information from many sensors is combined to yield an improved description of the observed system. In this section, we will give a brief introduction of the centralized square-root cubature information filter (CSCIF), which is the base of the proposed algorithm. A centralized camera network system comprises a fusion center with connections to all other cameras. In order to distinguish between the information state contribution *i* and node index, we use the *s* to denote the node index in the rest of paper. Each camera *C_s_* obtains data about the environment, which is forwarded to the fusion center, where *s* = (1, 2, ⋯ , *N_c_*). The global estimate in the fusion center can be computed from *N_C_* sensor measurements at time *k* by simple summing of the local information vectors and matrices (the “c” superscript denotes “centralized”):

(19) $$Y_{k|k}^{c}=Y_{k|k-1}^{c}+\sum_{s=1}^{N_{c}}I_{k}^{s}$$

(20) $${\hat{y}}_{k|k}^{c}={\hat{y}}_{k|k-1}^{c}+\sum_{s=1}^{N_{c}}i_{k}^{s}$$

In Equation (20), the $i_{k}^{s}$ can be computed using the equation $i_{k}^{s}=S_{i,k}^{s}{\bar{S}}_{R,k}^{s}(z_{k}^{s}-{\hat{z}}_{k|k-1}^{s})+S_{i,k}^{s}{\left(S_{i,k}^{s}\right)}^{T}{\hat{x}}_{k|k-1}^{s}$ [15], where $I_{k}^{s}=S_{i,k}^{s}{\left(S_{i,k}^{s}\right)}^{T}$, ${\bar{S}}_{R,k}^{s}={\left(S_{R,k}^{s}\right)}^{-T}$ and ${\left(R_{k}^{s}\right)}^{-1}={\bar{S}}_{R,k}^{s}{\left({\bar{S}}_{R,k}^{s}\right)}^{T}$. The square-root of $Y_{k|k}^{c}$ can be computed using Equation (17) as follows:

(21) $$S_{y,k|k}^{c}=\text{Tria}([\begin{array}{lllll} S_{y,k|k-1}^{c} & S_{i,k}^{1} & S_{i,k}^{2} & \cdots & S_{i,k}^{N_{c}} \end{array}])$$

Then, we compute the square-root of the predicted information matrix $S_{y,k+1|k}^{c}$ and the predicted information vector ${\hat{y}}_{k+1|k}^{c}$ using the standard time update step of SCIF.

Although the centralized camera network system is an improvement over a single camera system, it has a number of disadvantages. These include severe computational loads imposed on the fusion center, the possibility of catastrophic failure and high communication overheads.

### 4.3. Distributed Square-Root Cubature Information Weighted Consensus Filter

Generally, there are no fusion centers in large-scale camera networks, and the capabilities of all cameras are equal in the network. In this scenario, a distributed approach is required. The average consensus algorithm, which has been introduced in Section 3 of this paper, meets the needs of this scenario. In this section, we propose a novel DSCIWCF algorithm for target tracking in camera networks.

In the average consensus algorithm, node *C_s_* only communicates with its direct neighbors *C_j_* ∈ Ω_s_, then the values of the states at all of the nodes converge to the average of the initial values.

In [2], a distributed state estimation framework was proposed by Kamal *et al.* They used a value 1/*N_c_* as weights on the information matrix and information vector. This algorithm can overcome the naivety issue and information redundancy in camera networks. In this paper, we use a similar strategy to deal with the square-root cubature information matrix and information vector. Here, we summarize the DSCIWCF algorithm as follows.

(1) Compute the square-root form of the local information vector ${\hat{y}}_{k|k}^{s}$ and the information matrix $Y_{k|k}^{s}$ :
  (22) $$S_{y,k|k}^{s}=\text{Tria}([\begin{array}{cc} \frac{1}{\sqrt{N_{C}}}S_{y,k|k-1}^{s} & S_{i,k}^{s} \end{array}])$$
  (23) $${\hat{y}}_{k|k}^{s}=\frac{1}{N_{c}}{\hat{y}}_{k|k-1}^{s}+i_{k}^{s}$$
  (24) $$S_{i,k}^{s}=S_{y,k|k-1}^{s}{\left(S_{y,k|k-1}^{s}\right)}^{T}P_{s,xz,k|k-1}{\bar{S}}_{R,k}^{s}$$
  where $Y_{k|k-1}^{s}=S_{y,k|k-1}^{s}{\left(S_{y,k|k-1}^{s}\right)}^{T}$ and $P_{s,xz,k|k-1}=T_{21}T_{11}^{T}$ can be computed using Equation (A5) (see Appendix A). Equation (22) is equivalent to the equation below.
  (25) $$Y_{k|k}^{s}=\frac{1}{N_{c}}Y_{k|k-1}^{s}+I_{k}^{s}$$
(2) Let $\nu_{0}^{s}={\hat{y}}_{k|k}^{s}$ and $V_{0}^{s}=S_{y,k|k}^{s}$, then perform average consensus on $\nu_{0}^{s}$ and $V_{0}^{s}$ independently for *K* iterations:
  For *k* = 1 to *K*:
  Broadcast ( $\nu_{k-1}^{s}$, $V_{k-1}^{s}$) to neighbors *C*_*j*_, *C*_*j*_ ∈ Ω*_s_* and receive ( $\nu_{k-1}^{j}$, $V_{k-1}^{j}$) from neighbors.
  Run average consensus on $\nu_{k-1}^{s}$, $V_{k-1}^{s}$ :
  (26) $$V_{k}^{s}=\text{Tria}([\begin{array}{cccc} \sqrt{1-єN_{s,E}V_{k-1}^{s}} & \sqrt{є}V_{1,k-1}^{s} & \cdots & \sqrt{є}V_{N_{s,E},k-1}^{s} \end{array}])$$
  (27) $$\nu_{k}^{s}=\nu_{k-1}^{s}+є\sum_{j\in \Omega_{s}}\left(\nu_{k-1}^{j}-\nu_{k-1}^{s}\right)$$
  END for:
  where *N_s_*,*_E_* is the number of the direct neighbors of node *s*,*N*_*s*,*E*_ = Σ*_j_a_sj_*.
  If $V_{0}^{\prime s}=\frac{1}{N_{c}}Y_{k|k-1}^{s}+I_{k}^{s}$, the equivalent square form of the Equation (26) is as follows:
  (28) $$V_{k}^{\prime \text{s}}=V_{k-1}^{\prime s}+є\sum_{j\in \Omega_{s}}\left(V_{k-1}^{\prime j}-V_{k-1}^{\prime s}\right)$$
(3) Compute the *a posteriori* information vector and information matrix for time *k:*
  (29) $$\begin{array}{l} {\hat{y}}_{k|k}^{s}=N_{c}\nu_{K}^{s}\\ S_{y,k|k}^{s}=\sqrt{N_{c}}V_{K}^{s} \end{array}$$
(4) Compute the predicted information matrix $S_{y,k+1|k}^{s}$ and the predicted information vector ${\hat{y}}_{k+1|k}^{s}$ using the standard time update step of SCIF.

In practice, due to errors introduced by arithmetic operations (such as matrix square-root, matrix inversion, *etc*.) performed on finite word-length digital computers, the symmetry and positive definiteness of the error covariance matrix are often lost [9]. A square root filter is the best choice to deal with these problems. In this paper, we use the square-root cubature information filter for target tracking in camera networks. At the same time, we use Equations (26) and (27) for average consensus iterations. Therefore, the whole algorithm runs under the condition of the square-root filter.

### 5. Multi-Target Data Association

The JPDA algorithm has been introduced in Section 3. However, the traditional JPDA algorithm is often used for linear sensing models. In this section, we will extend the JPDA algorithm to handle nonlinear sensing models. The main contribution of this section is that we propose the algorithm derived from the combination of JPDA and the information filter mentioned in the last section. The JPDAF is a single sensor algorithm; thus, we firstly introduce the algorithm of the single node *s*.

#### 5.1. Joint Probabilistic Data Association With Square-Root Cubature Information Filter

In the SCIF, in order to make the information contribution equations compatible with those of the Kalman filter, a pseudo-measurement matrix $H_{s}^{t}$ [25] is defined as (at target *t*, similarly hereinafter):

(30) $$H_{s}^{t}=P_{s,xz,k|k-1}^{t^{T}}Y_{s,k|k-1}^{t}$$

where the subscript *s* denotes the terms from the *s*-th node.

In the cubature Kalman filter (CKF), the Kalman gain $K_{s,k}^{t}$ gives:

(31) $$K_{s,k}^{t}=P_{s,xz,k|k-1}^{t}{\left(P_{s,zz,k|k-1}^{t}\right)}^{-1}$$

where $K_{s,k}^{t}=T_{21}^{t}T_{11}^{t^{-1}}$ can be computed using Equation (A5) (see Appendix A).

The innovation covariance matrix is given by $W_{s}^{t}=H_{s}^{t}P_{s}^{t-}{\left(H_{s}^{t}\right)}^{T}+R_{s}^{t}$ [15], and substituting Equation (30) into the innovation covariance matrix, we can get:

(32) $$\begin{array}{ll} W_{s}^{t} & =P_{s,xz,k|k-1}^{t^{T}}Y_{s,k|k-1}^{t}P_{s,xz,k|k-1}^{t}+R_{s,k}^{t}\\ & =\Phi_{k|k-1}^{t}\Phi_{k|k-1}^{t^{T}}+R_{s,k}^{t}=P_{s,zz,k|k-1}^{t} \end{array}$$

where $Y_{s,k|k-1}^{t}={\left(P_{s,k|k-1}^{t}\right)}^{-1}$ is a symmetric positive definite matrix, and $P_{s,xz,k|k-1}^{t}$, $P_{s,zz,k|k-1}^{t}$, $P_{s,k|k-1}^{t}$ and $\Phi_{k|k-1}^{t}$ come from Equation (A1). $P_{s,zz,k|k-1}^{t}$ can be computed using $T_{11}^{t}T_{11}^{t^{T}}$ (see Appendix A). Now, we can rewrite Equation (31) as follows:

(33) $$K_{s,k}^{t}=P_{s}^{t-}{\left(H_{s}^{t}\right)}^{T}{\left(W_{s}^{t}\right)}^{-1}$$

where $H_{s,k}^{t}$ and $W_{s}^{t}$ are defined by Equations (30) and (32); $P_{s}^{t-}$ is the short form of $P_{s,k|k-1}^{t}$. In the CKF, the measurement innovation term of Equation (7) becomes,

(34) $${\tilde{z}}_{s}^{t}=z_{s}^{t}-(1-\beta_{i0}^{t}){\tilde{z}}_{s,k|k-1}$$

where ${\hat{z}}_{s,k|k-1}=\frac{1}{m}\sum_{j=1}^{m}Z_{sj,k|k-1}$ and *Z_sj,k_*_|_*_k−i_* denotes one of the propagated cubature points; see [15] for details about computing *Z_sj,k_*_|_*_k_*_−_*_i_*. Substituting Equation (34) into Equations (6) gives:

(35) $${\hat{x}}_{s}^{t+}={\hat{x}}_{s}^{t-}+K_{s,k}^{t}[z_{s}^{t}-(1-\beta_{s0}^{t}){\hat{z}}_{s,k|k-1}]$$

The JPDA state update Equation (35) has a similar form as the standard Kalman filter update, and it can be converted to the information form using $u_{s}^{t}=H_{s}^{t^{T}}R_{s}^{t^{-1}}z_{s}^{t}$ and $U_{s}^{t}=H_{s}^{t^{T}}R_{s}^{t^{-1}}H_{s}^{t}$. We get (see Appendix B):

(36) $${\hat{x}}_{s}^{t+}={\left(Y_{s}^{t-}+U_{s}^{t}\right)}^{-1}\{Y_{s}^{t-}{\hat{x}}_{s}^{t-}+u_{s}^{t}+U_{s}^{t}{\hat{x}}_{s}^{t-}-(1-\beta_{s0}^{t})H_{s}^{t^{T}}R_{s}^{t^{-1}}{\hat{z}}_{s,k|k-1}\}$$

where $R_{s}^{t}$ is the measurement noise covariance of node *s* for target *t* and $Y_{s}^{t-}={\left(P_{s}^{t-}\right)}^{-1}={\left(P_{s,k|k-1}^{t}\right)}^{-1}$.

The information matrix is denoted as follows (see Appendix B):

(37) $$Y_{s}^{t+}=Y_{s}^{t-}+G_{s}^{t}$$

where $G_{s}^{t}=Y_{s}^{t-}K_{s}^{t}{\left[{\left(M_{s}^{t}\right)}^{-1}-{\left(K_{s}^{t}\right)}^{T}Y_{s}^{t-}K_{s}^{t}\right]}^{-1}{\left(K_{s}^{t}\right)}^{T}Y_{s}^{t-}$, $M_{s}^{t}=(1-\beta_{s0}^{t})W_{s}^{t}-{\tilde{P}}_{s}^{t}$ and ${\tilde{P}}_{s}^{t}$ (see Equation (B4) in Appendix B). Equations (36) and (37) form the JPDA-SCIF algorithm.

#### 5.2. Joint Probabilistic Data Association With Centralized Square-Root Cubature Information Filter

In Equations (12) and (13), the information filter form has the advantage that the update equations for the estimator are computationally simpler than the equations for the Kalman filter. Here, we rewrite $I_{s}^{t}$ and $i_{s}^{t}$ using $H_{s}^{t}$ from Equation (30) as follows:

(38) $$I_{s}^{t}=H_{s}^{t^{T}}R_{s}^{t^{-1}}H_{s}^{t}$$

(39) $$i_{s}^{t}=H_{s}^{t^{T}}R_{s}^{t^{-1}}(z_{s}^{t}-{\hat{z}}_{s}^{t-}+H_{s}^{t}{\hat{x}}_{s,k|k-1}^{t})$$

where the measurement innovation term ${\tilde{z}}_{s}^{t}=z_{s}^{t}-{\hat{z}}_{s}^{t-}$ and ${\hat{x}}_{s,k|k-1}^{t}$ represents the prior/predicted state estimate. In the JPDAF, ${\tilde{z}}_{s}^{t}$ can be written as Equation (34). Therefore, Equation (39) can be rewritten as follows:

(40) $$i_{s}^{t}=H_{s}^{t^{T}}R_{s}^{t^{-1}}({\tilde{z}}_{s}^{t}+H_{s}^{t}{\hat{x}}_{s,k|k-1}^{t})$$

From Equations (38) and (40), we rewrite Equations (12) and (13) as follows:

(41) $$\begin{array}{ll} Y_{c}^{t+} & =Y_{c}^{t-}+\sum_{s=1}^{N_{C}}I_{s}^{t}\\ & =Y_{c}^{t-}+\sum_{s=1}^{N_{C}}H_{s}^{t^{T}}R_{s}^{t^{-1}}H_{s}^{t} \end{array}$$

(42) $$\begin{array}{ll} Y_{c}^{t+}{\hat{x}}_{c}^{t+} & =Y_{c}^{t-}{\hat{x}}_{c}^{t-}+\sum_{s=1}^{N_{C}}i_{s}^{t}\\ & =Y_{c}^{t-}{\hat{x}}_{c}^{t-}+\sum_{s=1}^{N_{C}}H_{s}^{t^{T}}R_{s}^{t^{-1}}({\tilde{z}}_{s}^{t}+H_{s}{\hat{x}}_{s}^{t-}) \end{array}$$

Substituting Equation (34) into Equation (42), we can extend Equations (36) and (37) into the multi-sensor centralized estimate in the information form as follows:

(43) $${\hat{x}}_{c}^{t+}={\left(Y_{c}^{t-}+\sum_{s=1}^{N_{C}}U_{s}^{t}\right)}^{-1}\{Y_{c}^{t-}{\hat{x}}_{c}^{t-}+\sum_{s=1}^{N_{C}}\left[u_{s}^{t}+U_{s}^{t}{\hat{x}}_{s}^{t-}-(1-\beta_{s0}^{t})H_{s}^{t^{T}}R_{s}^{t^{-1}}{\hat{z}}_{s,k|k-1}\}\right]$$

(44) $$Y_{c}^{t+}=Y_{c}^{t-}+\sum_{s=1}^{N_{C}}G_{s}^{t}$$

where $G_{s}^{t}=Y_{s}^{t-}K_{s}^{t}{\left[{\left(M_{s}^{t}\right)}^{-1}-{\left(K_{s}^{t}\right)}^{T}Y_{s}^{t-}K_{s}^{t}\right]}^{-1}{\left(K_{s}^{t}\right)}^{T}Y_{s}^{t-}$, $u_{s}^{t}=H_{s}^{t^{T}}R_{s}^{t^{-1}}z_{s}^{t}$, $U_{s}^{t}=I_{s}^{t}$, $M_{s}^{t}=(1-\beta_{s0}^{t})W_{s}^{t}-{\tilde{P}}_{s}^{t}$, and ${\tilde{P}}_{s}^{t}$ is defined as follows:

(45) $${\tilde{P}}_{s}^{t}=\sum_{j=1}^{m_{s}(k)}\beta_{sj}^{t}(z_{sj}-{\hat{z}}_{s,k|k-1}){\left(z_{sj}-{\hat{z}}_{s,k|k-1}\right)}^{T}-({\tilde{z}}_{s}^{t}){\left({\tilde{z}}_{s}^{t}\right)}^{T}$$

#### 5.3. Multi-Target Square-Root Cubature Information Weighted Consensus Filter

In the MTSCF, if all nodes have reached consensus on the previous time step, we will have ${\hat{x}}_{s}^{t-}={\hat{x}}_{c}^{t-}$ and $Y_{s}^{t-}=Y_{c}^{t-}$ for all *s*. That is to say, $Y_{c}^{t-}=\sum_{s=1}^{N_{C}}\frac{Y_{s}^{t-}}{N_{C}}$, and $Y_{c}^{t-}{\hat{x}}_{c}^{t-}=\sum_{s=1}^{N_{C}}\frac{Y_{s}^{t-}}{N_{C}}{\hat{x}}_{s}^{t-}$. Thus, we can write,

(46) $${\hat{x}}_{s}^{t+}=\sum_{s=1}^{N_{C}}{\left(\frac{Y_{s}^{t-}}{N_{C}}+U_{s}^{t}\right)}^{-1}\sum_{s=1}^{N_{C}}\left\{\frac{Y_{s}^{t-}}{N_{C}}{\hat{x}}_{s}^{t-}+u_{s}^{t}+U_{s}^{t}{\hat{x}}_{s}^{t-}-H_{s}^{t^{T}}R_{s}^{t^{-1}}(1-\beta_{s0}^{t}){\hat{z}}_{s,k|k-1}\right\}$$

(47) $$Y_{s}^{t+}\sum_{s=1}^{N_{C}}\left(\frac{Y_{s}^{t-}}{N_{C}}+G_{s}^{t}\right)$$

Thus, for the MTSCF algorithm, the consensus variables are initialized as,

(48) $$\nu_{s}^{t}[0]=\frac{Y_{s}^{t-}}{N_{C}}{\hat{x}}_{s}^{t-}+u_{s}^{t}+U_{s}^{t}{\hat{x}}_{s}^{t-}-(1-\beta_{s0}^{t})H_{s}^{t^{T}}R_{s}^{t^{-1}}{\hat{z}}_{s,k|k-1}$$

(49) $$V_{s}^{t}[0]=\frac{Y_{s}^{t-}}{N_{C}}+U_{s}^{t}$$

(50) $$M_{s}^{t}[0]=\frac{Y_{s}^{t-}}{N_{C}}+G_{s}^{t}$$

The MTSCF algorithm is summarized in Algorithm 1.

#### 5.4. Computing the Square-Root of the Information Matrix

The algorithms of this section that were mentioned above are based on information matrix. However, in order to be consistent with the algorithms proposed in this section, we need to transform the information matrix into the square-root form.

Illustrated by the case of the MTSCF algorithm, we will describe how to compute the square-root of the information matrix and other algorithms using similar methods.

Let $J_{s}^{t}J_{s}^{t^{T}}=M_{s}^{t}[0]$; because of the positive definite matrix of $M_{s}^{t}[0]$, we can use the Cholesky decomposition to compute $J_{s}^{t}$. The square-root form of Equation (50) is as follows:

(51) $$S_{M_{s}^{t}[0]}=\text{Tria}(J_{s}^{t})$$

It is easy to get $S_{U_{s}^{t}}$ and $S_{Y_{s}^{t-}}$; then, the square-root form of Equation (49) is as follows:

(52) $$S_{V_{s}^{t}[0]}=\text{Tria}([\begin{array}{cc} \frac{1}{\sqrt{N_{C}}}S_{Y_{s}^{t-}} & S_{U_{s}^{t}} \end{array}])$$

where $S_{Y_{s}^{t-}}$ and $S_{U_{s}^{t}}$ are the square-root form of $Y_{s}^{t-}$ and $U_{s}^{t}$, respectively. They can be computed using $U_{s}^{t}=S_{U_{s}^{t}}S_{U_{s}^{t}}^{T}$ and $Y_{s}^{t-}=S_{Y_{s}^{t-}}S_{Y_{s}^{t-}}^{T}$.


**Algorithm 1** MTSCF Algorithm for target *t* at node *C_s_* at time *k*.
**Input:**
$x_{s}^{t-}(k)$, 
$S_{Y_{s}^{t-}}(k)$ and 
$R_{s}^{t}$(1)Compute 
$H_{s}^{t}$ using Equation (30)(2)Get measurements: 
${\left\{z_{s}^{n}\right\}}_{n=1}^{m_{s}(k)}$(3)Compute 
$W_{s}^{t}$, 
$K_{s}^{t}$, 
$\beta_{s0}^{t}$, 
$M_{s}^{t}$, 
$z_{s}^{t}$, 
${\hat{z}}_{s,k|k-1}$, 
$Y_{s}^{t-}(k)$(4)Compute information vectors and matrices (using Equation (24)):
(53)$$u_{s}^{t}=H_{s}^{t^{T}}R_{s}^{t^{-1}}z_{s}^{t}$$
(54)$S_{U_{s}^{t}}=S_{Y_{s}^{t-}}(k)S_{Y_{s}^{t-}}{\left(k\right)}^{T}P_{s,xz,k|k-1}{\bar{S}}_{s,R,k}$(5)Broadcast message to neighbors and receive neighbors' messages(6)Compute inter-camera track-to-track matchings(7)Initialized consensus data
(55)$$\nu_{s}^{t}[0]=\frac{Y_{s}^{t-}}{N_{C}}{\hat{x}}_{s}^{t-}+u_{s}^{t}+U_{s}^{t}{\hat{x}}_{s}^{t-}-(1-\beta_{s0}^{t})H_{s}^{t^{T}}R_{s}^{t-1}{\hat{z}}_{s,k|k-1}$$
(56)$$S_{V_{s}^{t}[0]}=\text{Tria}([\begin{array}{cc} \frac{1}{\sqrt{N_{C}}}S_{Y_{s}^{t-}} & S_{U_{s}^{t}} \end{array}])$$
(57)$S_{M_{s}^{t}[0]}=\text{Tria}(J_{s}^{t})$(8)Perform average consensus on 
$\nu_{s}^{t}[0]$, 
$S_{V_{s}^{t}[0]}$ and 
$S_{M_{s}^{t}[0]}$ independently for *K* iterations(9)Estimate:
(58)$$\begin{array}{ll} {\hat{x}}_{s}^{t+}(k) & =\frac{1}{\left(S_{V_{s}^{t}[K]}S_{V_{s}^{t}[K]}^{T}\right)}\nu_{s}^{t}[K]\\ S_{Y_{s}^{t+}}(k) & =\sqrt{N_{c}}S_{M_{s}^{t}[K]} \end{array}$$(10)Compute the predicted state 
$S_{s,k+1|k}^{t}$ and the predicted error covariance 
${\hat{x}}_{s}^{t-}(k+1)$ [12], respectively, then compute the square-root of the predicted information matrix and information vector
(59)$$S_{Y_{s}^{t-}}(k+1)=S_{s,k+1|k}^{t^{-T}}$$**Output:**
${\hat{x}}_{s}^{t+}(k)$, 
$S_{Y_{s}^{t+}}(k)$, 
${\hat{x}}_{s}^{t-}(k+1)$, 
$S_{Y_{s}^{t-}}(k+1)$


#### 5.5. Inter-Camera Association

In distributed tracking of multiple targets, every node has a set of information from each of its neighbors about the targets and its own set of estimated tracks. Therefore, it is necessary to use an assignment algorithm to form a set of optimal matchings *g_sj_*, where *g_sj_* matches the tracks of node *s* with the tracks of node *j*. We can use the Hungarian algorithm [26] to find the maximum matching. The matching cost between two track estimates from different cameras can be defined as the Mahalanobis distance as follows [15]:

$$D({\bar{x}}_{s}^{t1},{\bar{x}}_{j}^{t2})={\left({\bar{x}}_{s}^{t1}-{\bar{x}}_{j}^{t2}\right)}^{T}{\left(P_{s}^{t1}+P_{j}^{t2}\right)}^{-1}({\bar{x}}_{s}^{t1}-{\bar{x}}_{j}^{t2})$$

### 6. Experimental Evaluation

In this section, we evaluate the performance of the proposed MTSCF algorithm in a nonlinear simulated environment and compare it with other methods: JPDA-EKCF [18] and EMTIC [20]. Our experiments are performed on an Intel 3.4 GHz PC with 4 G memory and implemented in MATLAB.

Four simulated targets (*N_T_* = 4) moving in a 500 m × 500 m area under the observation of nine cameras (*N_C_* = 9) with overlapping FOVs is considered. To simplify the simulation, the FOV of each camera is assumed to be a square region of 200 m × 200 m around the camera. The target's state vector is a 5D vector, which includes the target's position (*x_k_*, *y_k_*) at discrete time instant *k*, its velocity (*v_x_*, *v_y_*) and the time interval δ*_k_* between the two consecutive measurements. Accordingly, the state vector is given by *x_k_* = [*x_k_ y_k_ v_x_ v_y_* δ*_k_*]*^T^*.

The motion model of the targets is described by the nonlinear equation [8]:

(60) $${\text{X}}_{k+1}=\left(\begin{array}{c} x_{k}+v_{x,k}\delta_{k}+a_{x}\frac{\delta_{k}^{2}}{2}\\ y_{k}+v_{y,k}\delta_{k}+a_{y}\frac{\delta_{k}^{2}}{2}\\ v_{x,k}+a_{x}\delta_{k}\\ v_{y,k}+a_{y}\delta_{k}\\ \delta_{k}+e \end{array}\right)$$

where the target acceleration (a_x_, a_y_) is modeled as Gaussian noise. To account for synchronization errors among cameras, we consider a time uncertainty *e*, which is also assumed to be a Gaussian variable. We consider the vector *v* = (*a_x_*, *a_y_*, *e*) as the Gaussian noise vector with zero mean and covariance *Q* = *diag*([5 5 0.01]). The initial speed is randomly obtained from the range 10 ˜ 30 units per time step and with a random direction uniformly chosen from 0−2π.

The measurement model can be defined as:

(61) $${\text{z}}_{k}^{s}=\left(\begin{array}{c} \gamma_{k}^{s}\\ \phi_{k}^{s} \end{array}\right)=\left(\begin{array}{c} \frac{H_{11}^{s}x_{k}+H_{12}^{s}y_{k}+H_{13}^{s}}{H_{31}^{s}x_{k}+H_{32}^{s}y_{k}+H_{33}^{s}}\\ \frac{H_{21}^{s}x_{k}+H_{22}^{s}y_{k}+H_{23}^{s}}{H_{31}^{s}x_{k}+H_{32}^{s}y_{k}+H_{33}^{s}} \end{array}\right)+w_{k}$$

where ( $\gamma_{k}^{s}$, $\phi_{k}^{s}$) is the pixel coordinates of the target in the image plane of camera *C_s_* at time *k*. The values $H_{11}^{s}$, … , $H_{33}^{s}$ are the elements of homography; *w_k_* is the measurement noise, which is considered to be Gaussian with zero mean and variance *R* = *diag*([5 5]). The homography matrix values of each camera are taken from the camera *C*_6_ of the APIDISdataset [27] whose values are:

(62) $$H_{s}=\left(\begin{array}{ccc} 1,930.8939 & -89.8033 & -2,393,800\\ 117.2530 & 91.8121 & 1,022,700\\ 0.3485 & -0.8720 & 1,971.8862 \end{array}\right)$$

The initial prior covariance $P_{s}^{t-}(1)=\text{diag}([100,100,10,10,0.01])$ is used at each node for each target. The initial prior state ${\hat{x}}_{s}^{t-}\left(1\right)$ is generated by adding zero-mean Gaussian noise of covariance $P_{s}^{t-}\left(1\right)$ to theinitial ground truth state. The observations are generated using Equation (61). The total number of consensus iterations per measurement step, *K*, is set to 20. The parameters for computing the association probabilities, $\beta_{sj}^{t}$, are set as follows (see [14] for details about computing $\beta_{sj}^{t}$). False measurements (clutter) are generated at each node at each measurement step using a Poisson process with λ, where λ is the average number of false measurements per sensor per measurement step. Gate probability *P_G_* is set to 0.99. The probability of detecting a target *P_D_* in each camera is set to 0.8.

**Figure 1:**
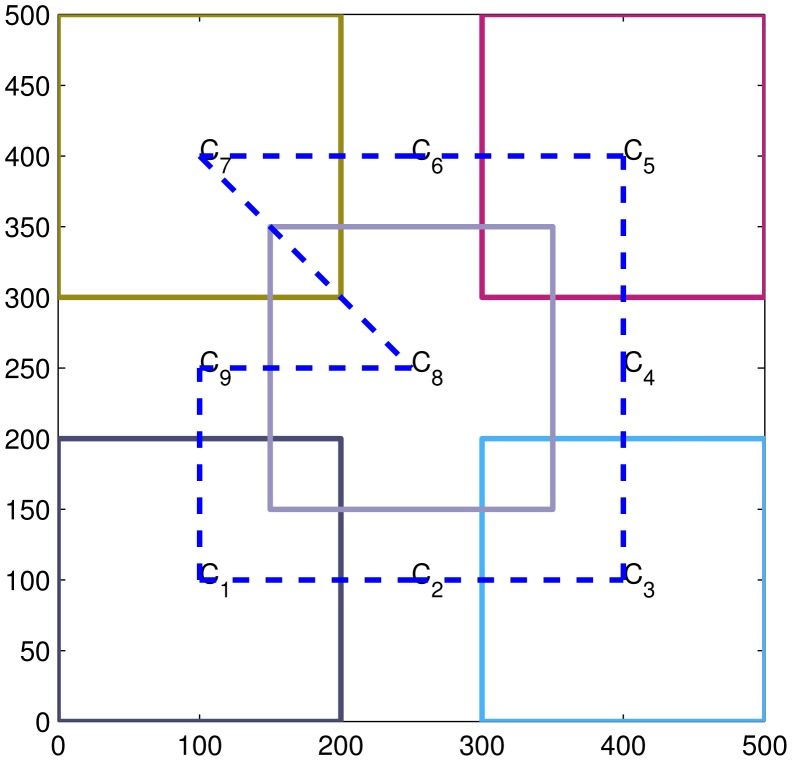
Sparse connectivity of the network.

**Figure 2:**
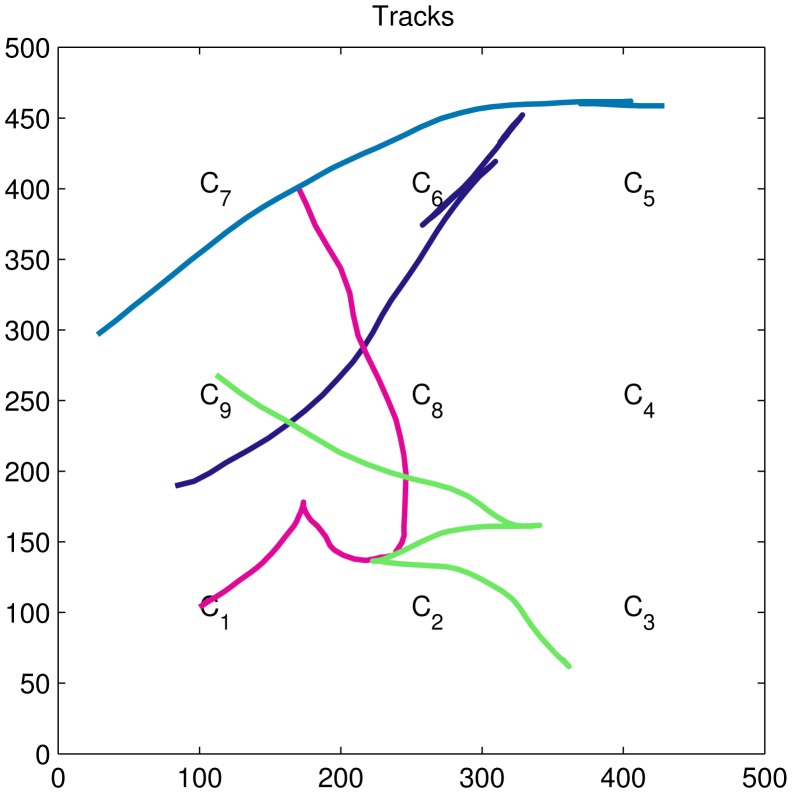
Four true trajectories of targets and their geographical positions in the cameras.

In this paper, we perform the experiments for a sparse connectivity network with a low average network degree equal to two (see Figure 1). Therefore, the Δ*_max_* = 2; then, the consensus rate parameter є is set to 0.65/Δ*_max_*. In the experiment, four targets' trajectories are generated (see Figure 2). The simulation results are averaged over 20 Monte Carlo simulation runs.

**Figure 3:**
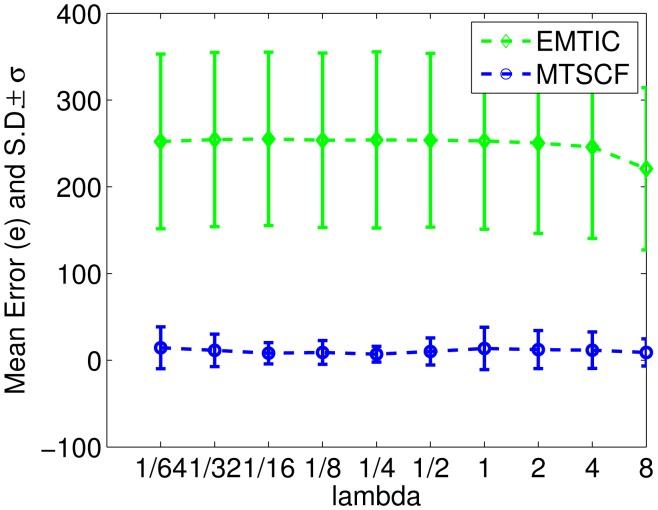
Performance comparison by varying the amount of clutter.

Figure 3 shows the performance comparison by varying the amount of clutter. The average amount of clutter per sensor per measurement step λ is varied from 1/64−8 (consensus is run for a fixed number of iterations (eight)). From Figure 3, it can be seen that both EMTIC and MTSCF are very robust, even to a very high amount of clutter. The amount of clutter is kept at λ = 1 for the other experiments in the rest of the paper.

**Figure 4:**
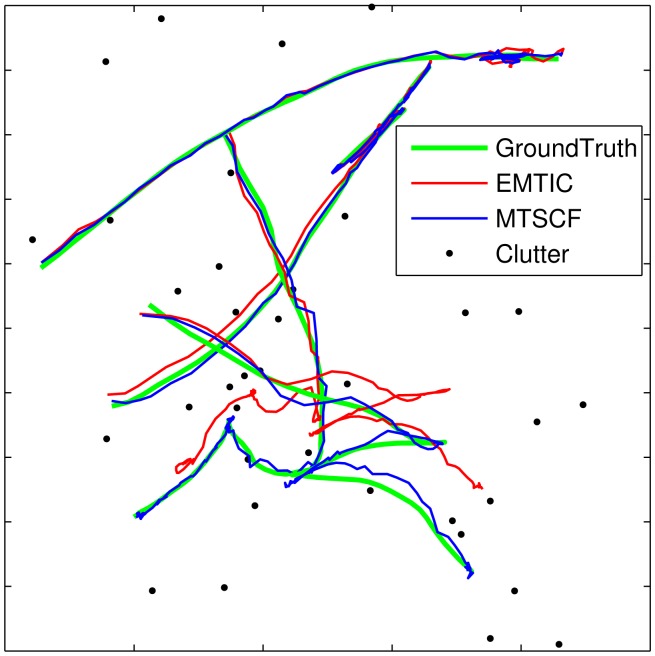
The result of target tracking in one experiment from one run.

The result of target tracking can be seen in Figure 4 in one experiment from one run (the result is based on the consensus algorithm, and the number of consensus iterations is eight). As you can see from Figure 4, the MTSCF algorithm is closer to the ground-truth curves than EMTIC.

**Figure 5:**
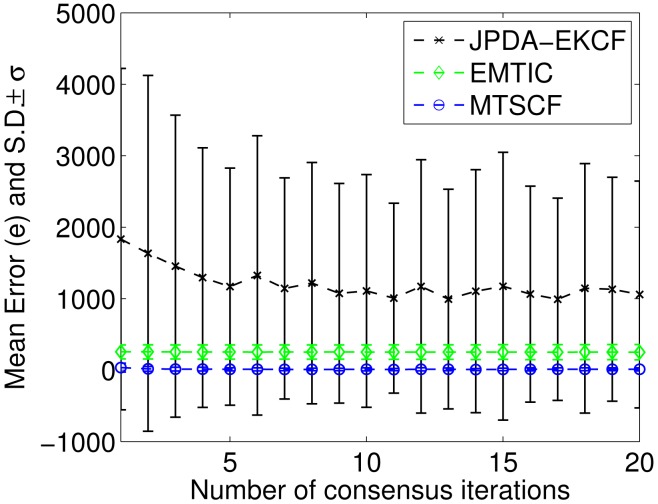
The mean errors and the variation of the estimation errors about three algorithms (joint probabilistic data association (JPDA)-EKCF, EMTICand multi-target square-root cubature information weighted consensus filter (MTSCF)).

**Figure 6:**
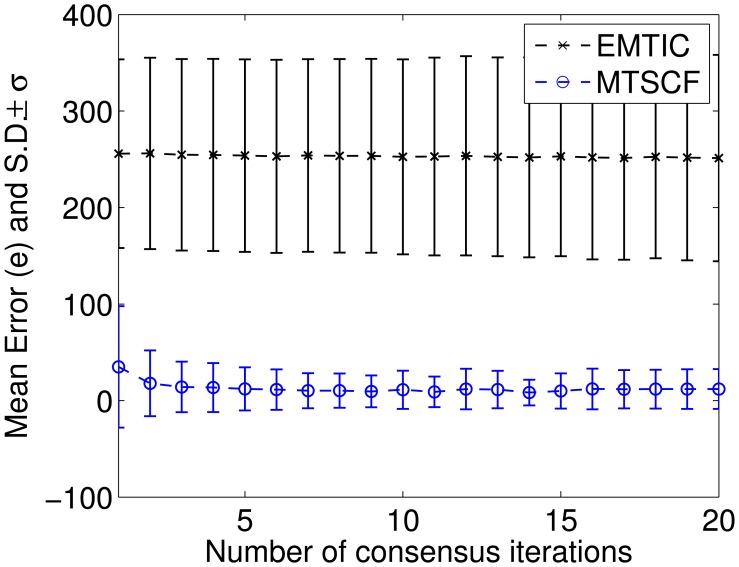
Zoom of Figure 5 with focus on MTSCF and EMTIC.

To show the convergence of different methods, the total number of iterations per measurement step, *K*, is varied. It can be seen from Figures 5 and 6 (Figure 6 shows an enlarged part of Figure 5 focusing on MTSCF and EMTIC) that with an increased number of iteration, MTSCF approaches the ground-truth tracks. It can also be seen that MTSCF outperforms EMTIC for any given *K*. Meanwhile, It can be seen that JPDA-EKCF has large mean error, which does not suit nonlinear multi-target tracking in distributed camera networks.

Our simulation is based on MATLAB. In MATLAB, it handles floating-point numbers in double precision (default setting) format; while double precision numbers use 64 bits, based on IEEE Standard 754. In the experiment, we convert all double precision numbers to single precision (32 bits) numbers using the command single (number). Unfortunately, it may be impossible for us to use the single precision in JPDA-EKCF and EMTIC. The reason is that, when the single precision number is used to calculate the updated inverse matrix, the resulting matrix may possibly be non-positive definite. In the simulation, we get the warning “Matrix is close to singular or badly scaled. Results may be inaccurate.” Hence, errors may occur during the execution of the JPDA-EKCF and EMTIC algorithms in a limited word-length system. This is not a problem for the MTSCF algorithm.

## 7. Conclusions

In this paper, we propose a novel multi-target square-root cubature information weighted consensus filter (MTSCF) algorithm, which is a generalized consensus-based distributed multi-target tracking scheme applicable to a wide variety of sensor networks. MTSCF handles the issue of naivety, which makes it applicable to sensor networks where sensors may have limited FOV (which is the case for a camera network). The algorithm is efficient for considering the estimation errors in tracking and data association, the influence of naivety and the numerical difficulties from the finite word-length of computers, which makes it resistive to false measurements/clutter. Experimental analysis shows the strength of the proposed method over existing ones.

In our future work, we will explore applying the MTSCF to a real camera network, which may be a limited word-length embedded system. Handling out-of-sequence measurements, the unknown number of targets and asynchronous networks are some other possible future works.

## Appendixes

### A. Squared-Matrix Forms of Three Covariance Matrices

*P_k_*_|_*_k_*_−1_, *P_zz,k_*_|_*_k_*_−1_, *P_xz,k_*_|_*_k_*_−1_ may be expressed in squared-matrix forms [28]:

(A1) $$\left(\begin{array}{cc} P_{zz,k|k-1} & P_{zx,k|k-1}\\ P_{xz,k|k-1} & P_{k|k-1} \end{array}\right)=\left(\begin{array}{cc} \Phi_{k|k-1} & S_{R,k}\\ \Psi_{k|k-1} & 0 \end{array}\right){\left(\begin{array}{cc} \Phi_{k|k-1} & S_{R,k}\\ \Psi_{k|k-1} & 0 \end{array}\right)}^{T}$$

where *S_R,k_* is the square-root of *R_k_*, 0 ∈ **R***^n_x_^*^×^*^n_z_^*, and:

(A2) $$\begin{array}{cccc} \Psi_{k|k-1}=\frac{1}{\sqrt{m}}[X_{1,k|k-1}-{\hat{x}}_{k|k-1} & X_{2,k|k-1}-{\hat{x}}_{k|k-1} & \cdots & X_{m,k|k-1}-{\hat{x}}_{k|k-1}] \end{array}$$

(A3) $$\begin{array}{cccc} \Phi_{k|k-1}=\frac{1}{\sqrt{m}}[Z_{1,k|k-1}-{\hat{z}}_{k|k-1} & Z_{2,k|k-1}-{\hat{z}}_{k|k-1} & \cdots & Z_{m,k|k-1}-{\hat{z}}_{k|k-1}] \end{array}$$

Applying the triangularization procedure to the square-root factor available on the RHS of Equation (A1) yields:

(A4) $$\text{Tria}\left(\begin{array}{cc} \Phi_{k|k-1} & S_{R,k}\\ \Psi_{k|k-1} & 0 \end{array}\right)=\left(\begin{array}{cc} T_{11} & 0\\ T_{21} & T_{22} \end{array}\right)$$

where *T*_11_ ∈ **R***^n_z_^*^×^*^n_z_^*, *T*_22_ ∈ **R***^n_x_^*^×^*^n_x_^* are lower triangular matrices, and *T*_21_ ∈ **R***^n_x_^*^×^*^n_z_^*. Equation (A1) can be rewritten as follows:

(A5) $$\begin{array}{l} \left(\begin{array}{cc} P_{zz,k|k-1} & P_{zx,k|k-1}\\ P_{xz,k|k-1} & P_{k|k-1} \end{array}\right)\\ =\left(\begin{array}{cc} T_{11} & 0\\ T_{21} & T_{22} \end{array}\right){\left(\begin{array}{cc} T_{11} & 0\\ T_{21} & T_{22} \end{array}\right)}^{T}\\ =\left(\begin{array}{cc} T_{11}T_{11}^{T} & T_{11}T_{21}^{T}\\ T_{21}T_{11}^{T} & T_{21}T_{21}^{T}+T_{22}T_{22}^{T} \end{array}\right) \end{array}$$

### B. JPDA-SCIF: Derivation

Kalman gain can be rewritten as follows [20]. The time index *k* has been dropped for simplicity.

(B1) $$\begin{array}{ll} K_{s} & =P_{s}^{t-}{\left(H_{s}^{t}\right)}^{T}{\left(W_{s}^{t}\right)}^{-1}\\ & ={\left(P_{s}^{t{-}^{-1}}+H_{s}^{t^{T}}R_{s}^{t{-}^{-1}}H_{s}^{t}\right)}^{-1}H_{s}^{t^{T}}R_{s}^{t{-}^{-1}} \end{array}$$

The state estimate,

(B2) $$\begin{array}{ll} {\hat{x}}_{s}^{t+} & ={\hat{x}}_{s}^{t-}+K_{s}^{t}{\tilde{z}}_{s}^{t}\\ & ={\hat{x}}_{s}^{t-}+K_{s}^{t}[z_{s}^{t}-(1-\beta_{s0}^{t}){\hat{z}}_{s,k|k-1}]\\ & ={\hat{x}}_{s}^{t-}+{\left(P_{s}^{t{-}^{-1}}+H_{s}^{t^{T}}R_{s}^{t^{-1}}H_{s}^{t}\right)}^{-1}H_{s}^{t^{T}}R_{s}^{t^{-1}}[z_{s}^{t}-(1-\beta_{s0}^{t}){\hat{z}}_{s,k|k-1}]\\ & ={\hat{x}}_{s}^{t-}+{\left(Y_{s}^{t-}+U_{s}^{t}\right)}^{-1}(u_{s}^{t}-(1-\beta_{s0}^{t})H_{s}^{t^{T}}R_{s}^{t^{-1}}{\hat{z}}_{s,k|k-1})\\ & ={\left(Y_{s}^{t-}+U_{s}^{t}\right)}^{-1}\{Y_{s}^{t-}{\hat{x}}_{s}^{t-}+u_{s}^{t}+U_{s}^{t}{\hat{x}}_{s}^{t-}-(1-\beta_{s0}^{t})H_{s}^{t^{T}}R_{s}^{t^{-1}}{\hat{z}}_{s,k|k-1}\} \end{array}$$

For the covariance, rewrite Equation (8) as follows:

(B3) $$\begin{array}{ll} P_{s}^{t+} & =P_{s}^{t-}-(1-\beta_{s0}^{t})K_{s}^{t}W_{s}^{t}{\left(K_{s}^{t}\right)}^{T}+K_{s}^{t}{\tilde{P}}_{s}^{t}{\left(K_{s}^{t}\right)}^{T}\\ & =P_{s}^{t-}-K_{s}^{t}[(1-\beta_{s0}^{t})W_{s}^{t}-{\tilde{P}}_{s}^{t}{\left(K_{s}^{t}\right)}^{T} \end{array}$$

where,

(B4) $${\tilde{P}}_{s}^{t}=\sum_{j=1}^{m_{s}(k)}\beta_{sj}^{t}(z_{sj}-{\hat{z}}_{s,k|k-1}){\left(z_{sj}-{\hat{z}}_{s,k|k-1}\right)}^{T}-\left({\tilde{z}}_{s}^{t}\right){\left({\tilde{z}}_{s}^{t}\right)}^{T}$$

Let $M_{s}^{t}=(1-\beta_{s0}^{t})W_{s}^{t}-{\tilde{P}}_{s}^{t}$, then use the matrix inversion lemma on Equation (B3); we get:

(B5) $$Y_{s}^{t+}=Y_{s}^{t-}+G_{s}^{t}$$

where $G_{s}^{t}=Y_{s}^{t-}K_{s}^{t}{\left[{\left(M_{s}^{t}\right)}^{-1}-{\left(K_{s}^{t}\right)}^{T}Y_{s}^{t-}K_{s}^{t}\right]}^{-1}{\left(K_{s}^{t}\right)}^{T}Y_{s}^{t-}$.
